# Protein Tyrosine Phosphatase Receptor Zeta 1 as a Potential Target in Cancer Therapy and Diagnosis

**DOI:** 10.3390/ijms24098093

**Published:** 2023-04-30

**Authors:** Evangelia Papadimitriou, Vasiliki K. Kanellopoulou

**Affiliations:** Laboratory of Molecular Pharmacology, Department of Pharmacy, University of Patras, 26504 Patras, Greece

**Keywords:** angiogenesis, cancer, endothelial cells, PTPRZ1, tyrosine phosphatase, tyrosine phosphorylation

## Abstract

Protein tyrosine phosphatase receptor zeta 1 (PTPRZ1) is a type V transmembrane tyrosine phosphatase that is highly expressed during embryonic development, while its expression during adulthood is limited. PTPRZ1 is highly detected in the central nervous system, affecting oligodendrocytes’ survival and maturation. In gliomas, PTPRZ1 expression is significantly upregulated and is being studied as a potential cancer driver and as a target for therapy. PTPRZ1 expression is also increased in other cancer types, but there are no data on the potential functional significance of this finding. On the other hand, low PTPRZ1 expression seems to be related to a worse prognosis in some cancer types, suggesting that in some cases, it may act as a tumor-suppressor gene. These discrepancies may be due to our limited understanding of PTPRZ1 signaling and tumor microenvironments. In this review, we present evidence on the role of PTPRZ1 in angiogenesis and cancer and discuss the phenomenal differences among the different types of cancer, depending on the regulation of its tyrosine phosphatase activity or ligand binding. Clarifying the involved signaling pathways will lead to its efficient exploitation as a novel therapeutic target or as a biomarker, and the development of proper therapeutic approaches.

## 1. Introduction

Protein tyrosine phosphorylation is a post-translational modification that has a significant role during development, as well as in several pathologies, among which are inflammation and cancer. The balance between the action of tyrosine kinases (TKs) and tyrosine phosphatases (TPs) is significant for homeostasis and impaired in pathologies and has been the focus of numerous studies that have led to TK inhibitors (TKIs), used primarily in cancer therapy. TPs have not been extensively studied to date and are subdivided into the transmembrane receptor protein TPs and the non-receptor intracellular TPs. There are also dual-specificity phosphatases and low molecular weight phosphatases that are even less extensively studied [1].

In the present review, we focus on the involvement of the transmembrane protein tyrosine phosphatase receptor zeta 1 (PTPRZ1) in cancer growth and angiogenesis and discuss its potential as a drug target or as a biomarker, depending on the tumor type.

## 2. Structure of PTPRZ1 Protein

PTPRZ1 belongs to the type V transmembrane TPs and has three known splicing variants: PTPRZ-A, which is a full-length receptor form; PTPRZ-B, which is a shorter receptor form that has a deletion in the extracellular region compared to PTPRZ-A, and PTPRZ-S, which is a secretory variant of PTPRZ-A known as 6B4 proteoglycan/phosphacan. The transmembrane isoforms share common structural characteristics, such as an extracellular carbonic anhydrase-like domain at the N-terminal of the receptor, followed by a fibronectin type III (FNIII)-like domain, a transmembrane region, two TP catalytic domains, and a C-terminal PDZ-binding motif. The shorter PTPRZ-B isoform lacks exon 12, which encodes 860 amino acids in the extracellular domain following the FNIII domain and forms a Ser-Gly-rich region for glycosaminoglycan attachment. This domain can be heavily glycosylated in PTPRZ-A and phosphacan. In all isoforms, the TP catalytic domain closer to the membrane (D1) is active, whereas the TP domain farther from the membrane (D2) is inactive [1]. More PTPRZ1 isoforms have been identified that differ in the N-terminal region of the membrane-proximal TP domain, their affinity to postsynaptic density protein 95, and their catalytic efficiency in vitro [2]. The distribution of these isoforms and any functional consequences remain unknown.

Glycosylation of PTPRZ1 varies between tissues, at different developmental stages, or in pathologies. In the central nervous system, PTPRZ1 is highly modified by chondroitin sulfate (CS), keratan sulfate, and human natural killer-1 (HNK-1) carbohydrates. In other tissues, such as gastric glands, PTPRZ1 seems to lack glycosylation [3,4]. The significance of PTPRZ1 glycosylation lies in the fact that it affects the binding of ligands, such as pleiotrophin (PTN), midkine (MK), and interleukin 34 (IL34) [5,6,7,8,9]. PTN may also bind to the protein core of PTPRZ1 independently of the CS moieties [10,11], at a site that has not been identified to date.

## 3. Regulation of PTPRZ1 Expression

PTPRZ1 expression is high in embryonic stem cells, while in the adult its expression is low, except in the nervous system [12]. In cancer, PTPRZ1 expression is upregulated or downregulated, as discussed later in this review, but how this is moderated in each case has not been elucidated.

In general, studies related to the regulation of PTPRZ1 expression are few and superficial. It has been shown in astrocytes, for example, that epidermal growth factor (EGF) and transforming growth factor alpha (TGFα) strongly increase the expression of both transmembrane PTPRZ1 isoforms and phosphacan through the EGF receptor, while interferon-gamma and tumor necrosis factor-alpha (TNFα) decrease the expression of phosphacan, but not of the transmembrane PTPRZ1 isoforms. TGFβ1 causes a small upregulation, while fibroblast growth factor 2 (FGF2), IL1, IL6, ciliary neurotrophic factor, leukemia inhibitory factor, and platelet-derived growth factor have no effect [13]. The small, leucine-rich proteoglycan decorin significantly decreases the protein expression of phosphacan in a mouse model of acute spinal cord injuries [14], and PTN positively regulates PTPRZ1 expression in glioma and breast cancer cells [8,15]. Chronic oxidative stress induces genomic amplification of the *Ptprz1* gene in renal cell carcinoma cells [16], while doxorubicin has been shown to upregulate PTPRZ1 expression in triple-negative breast cancer cells [15]. The signaling and/or the transcription factors involved in these effects are not known to date.

PTPRZ1 expression increased after hypoxia-inducible factor (HIF) 2 but not HIF1 vector transfection in HEK293T cells, suggesting that the *Ptprz1* gene may be a target of hypoxia [17]. It was later found that the *Ptprz1* promoter contains HIF- and E26 transformation-specific (Ets)-binding motifs, and the preferential activation of *Ptprz1* by HIF2, but not HIF1, may derive from the cooperative binding of HIF2 and Ets Like-1 (ELK1) to the nearby corresponding sites on the *Ptprz1* promoter [18]. The tumor-suppressor von Hippel-Lindau (VHL) gene, which plays an important role in regulating the response to hypoxia, suppresses PTPRZ1 expression [19], potentially by suppressing HIF2 expression. The PPARα agonist clofibrate downregulates the expression of PTPRZ1 in pancreatic cancer cells, by abrogating the binding of nuclear factor-κB (NFκΒ) to the *Ptprz1* promoter [20]. More recently, HOXA5 has been shown to directly bind to the *Ptprz1* promoter and upregulate its expression, resulting in increased glioma malignancy [21].

## 4. Interaction of PTPRZ1 with Cellular and Soluble Ligands

One of the reasons why TPs are left behind in the race of tyrosine phosphorylation studies is that their ligands are mostly unknown. PTPRZ1 is one of the few TPs with identified soluble ligands, which has sped up research related to its pharmacological targeting.

The initial efforts to identify PTPRZ1-binding molecules investigated cellular proteins that interacted with the soluble PTPRZ1 isoform phosphacan in the nervous system. Those early studies showed that the extracellular PTPRZ1 domain interacts with the neural cell adhesion molecule (N-CAM) and the neuron-glia CAM (Ng-CAM), but not with extracellular matrix proteins. Low levels of interaction were seen only with collagens I, II, VI, and tenascin [22]. Specific and high-affinity interaction of tenascin with phosphacan was also found in another study [23]. These interactions seem to be mediated by asparagine-linked oligosaccharides present in the carbonic anhydrase- and FNIII-like domains of PTPRZ1/phosphacan [24,25]. Another neuronal cell adhesion protein, contactin, interacts with the carbonic anhydrase domain of PTPRZ1 and may generate unidirectional or bidirectional signals during neural development [26]. Another CAM of the Ig superfamily, F3 on neurons, has also been found to interact with PTPRZ1 on Schwann cells, affecting peripheral nerve development and regeneration [27]. In brain neurons, besides cell adhesion molecules, the short-transmembrane PTPRZ1 isoform and phosphacan have been found to associate with voltage-gated sodium channels through their carbonic anhydrase domain and affect sodium ion currents [28].

More recently, α_4_β_1_, α_6_β_1_, and α_ν_β_3_ integrins were found to directly interact with PTPRZ1 in normal and cancer cells [29,30,31]. The interaction of α_ν_β_3_ with PTPRZ1 in endothelial cells is independent of the presence of any ligand [32]. PTPRZ1 also interacts with the low-density lipoprotein receptor-related protein-6 (LRP6) ectodomain [29] and with nucleolin [33], but mechanistic aspects, or the functional significance of these interactions, remain unknown.

The first PTPRZ1-soluble ligand was identified in an extract of brain microsomal fractions and was PTN [5]. A few years later, MK, which belongs to the same protein family as PTN, was also found to bind PTPRZ1 [6]. Both PTN and MK have low- and high-affinity binding sites on PTPRZ1 and at least one of the binding sites depends on PTPRZ1 glycosylation [5,6,10,11]. The PTN or MK domains that bind to PTPRZ1 have not been identified. The carboxyterminal unstructured domain of PTN has been suggested to mediate this interaction [11], although this has not been verified in all types of cells [34] and this domain has no similarity between PTN and MK. In MK, Arg78 at its C-terminal thrombospondin type 1 repeat (TSR) domain plays an essential role in high-affinity binding to CS moieties on PTPRZ1 [6].

FGF2 has been early identified as a PTPRZ1 ligand which binds PTPRZ1 with high affinity. Binding was not affected by the chondroitinase treatment of phosphacan, suggesting that it takes place on the protein core of the receptor [35]. However, this interaction and its potential functional significance in the nervous or other systems, physiology, or pathology, has not been studied further.

The *Helicobacter pylori* exotoxin VacA, which is responsible for gastric injury, has also been identified as a PTPRZ1 ligand. VacA interacts with the PTPRZ1 protein core sequence QTTQP at positions 747–751, which is distinct from the PTN-binding site(s) [36].

In studies aiming at identifying how IL34 is active in the absence of its main receptor, the colony-stimulating factor 1 receptor, PTPRZ1 has been identified as a functional receptor for IL34. IL34 binds to PTPRZ1 in a CS-dependent manner, at a site or sites that are different from the PTN-binding sites, based on the observation that PTN does not compete with IL34 for PTPRZ1 binding [8].

The most recently identified soluble ligand for PTPRZ1 is vascular endothelial growth factor A (VEGFA). VEGFA competes with PTN for PTPRZ1 binding, and both PTN and VEGFA seem to activate similar signaling downstream of the receptor. The monoclonal antibody bevacizumab, which binds to VEGFA and inhibits its binding to its VEGF receptors 1 and 2, does not affect binding to PTPRZ1, suggesting that the VEGF receptor-binding domain of VEGFA is not involved in PTPRZ1 binding. The heparin-binding domain of VEGFA is also not involved [37,38], and the VEGFA domain that interacts with PTPRZ1 is still to be identified.

Finally, it has recently been shown by using the RNA-chromatin immunoprecipitation assay that the long non-coding RNA (lncRNA) uveal melanoma formation-transcript 1 (OUM1) functions by directly binding to the PTPRZ1 protein in the cytoplasm of uveal melanoma cells, to enhance its TP activity [39], highlighting a novel pathway of PTPRZ1 regulation.

**Figure 1:**
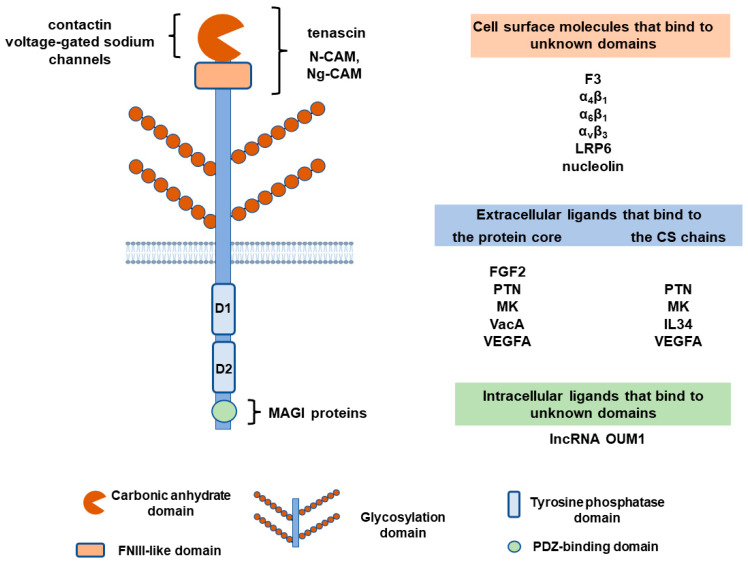
Schematic representation of the PTPRZ1 extracellular and intracellular domains and molecules that have been shown to interact with it. In some cases, such as CAMs and tenascin, the interacting domain has been identified, while for others, such as integrins, it remains unknown. Soluble ligands may interact either with the protein core or with the CS moieties of the long PTPRZ1 isoform. In all cases, the involved protein core domain of PTPRZ1 has not been identified. For more details, see the text.

Figure 1 summarizes knowledge of the molecules that interact with PTPRZ1.

## 5. Signaling Downstream of PTPRZ1

Following PTN’s identification as the first PTPRZ1-soluble ligand, it was suggested that PTN binding to the extracellular domain of the receptor in U373-MG cells leads to its dimerization and inactivation of its TP activity [40,41]. The result of this inactivation has been the tyrosine phosphorylation of β-catenin, resulting in an impaired association between β-catenin and Ν-cadherin, disrupted cell-to-cell adhesion complexes, initiation of the epithelial-to-mesenchymal transition (EMT), and tumor development and progression [40,41]. In fetal human oligodendrocyte progenitor cells, exogenous PTN or *Ptprz1* gene deletion strongly potentiated cell proliferation mediated by the tonic activation of β-catenin/T-cell factor-dependent transcription [42]. In renal cell carcinoma cell lines, β-catenin has been found to co-immunoprecipitate with PTPRZ1, and downregulation of PTPRZ1 by siRNA led to increased tyrosine phosphorylation of β-catenin and a significant decrease of nuclear β-catenin, leading to decreased cell proliferation [16]. However, in another study in human renal cell carcinoma cells, PTPRZ1 was shown to increase nuclear β-catenin protein levels and enhance cell proliferation [19]. Overexpression of PTPRZ1 promotes the malignant transformation of oral submucous fibrosis, at least partly, through its effect on β-catenin phosphorylation [43]. In human umbilical vein endothelial cells (HUVEC), tyrosine phosphorylation or localization of β-catenin was unaltered following PTN stimulation [44].

Beta (β)-adducin was also found to interact with the intracellular PTPRZ1 domain in a yeast two-hybrid system. In Hela cells stimulated by PTN, β-adducin phosphorylation is increased, leading to disrupted actin/spectrin/β-adducin complexes, and loss of stabilized cytoskeleton and homophilic cell–cell adhesion [45]. Activation of the PTN/PTPRZ1 signaling pathway has also been shown to induce calmodulin phosphorylation in small-cell lung cancer cells [46].

Using the yeast two-hybrid system, the Src TK family member Fyn was found to interact with the intracellular domain of PTPRZ1 [47], while more recently, this interaction was verified in glioblastoma (GBM) stem cells [48]. PTN binding to PTPRZ1 in Hela [47] and GBM stem cells [48] leads to a sharp increase in the tyrosine phosphorylation of Fyn, suggesting that Fyn is a PTPRZ1 substrate. However, in the spinal cord of *Ptprz1*-deficient C57BL/6 mice with experimental autoimmune encephalomyelitis [49], and in mouse oligodendrocyte-lineage OL1 cells [50], it was shown that there were no significant differences in the tyrosine phosphorylation of Fyn related to PTPRZ1. Another member of the same TK family, c-Src, has also been found to co-immunoprecipitate with PTPRZ1 [51] and is activated upon PTN binding to PTPRZ1 in HUVEC [33,37,51].

The GTPase-activating protein (GAP) for Rho GTPase, p190 Rho GAP, has also been shown to be a PTPRZ1 substrate based on the observations that: (a) in *Ptprz1*-deficient C57BL/6 mice, phosphorylation of Tyr1105 on p190 Rho GAP is not affected by fear conditioning, while in the corresponding wildtype mice, it was decreased, and (b) PTN increases phosphorylation of p190 Rho GAP at Tyr1105 in B103 neuroblastoma cells in a PTPRZ1-dependent manner [52]. In the spinal cord of *Ptprz1*-deficient C57BL/6 mice with experimental autoimmune encephalomyelitis, Tyr1105 phosphorylation in p190 Rho GAP was independent of Fyn, supporting the notion that p190 Rho GAP is a PTPRZ1 substrate [49,53]. PTPRZ1-dependent and Fyn-independent p190 Rho GAP Tyr1105 phosphorylation has also been shown in mouse oligodendrocyte-lineage OL1 cells [50].

The neuregulin receptor ErbB4, which is a member of the ErbB-family TKs, has been found to functionally interact with the intracellular carboxyl-terminal region of PTPRZ1 in adult rat synaptosomes, and in cell bodies and apical dendrites of neurons in the prefrontal cortex. Tyrosine phosphorylation of ErbB4 is increased in *Ptprz1*-deficient C57BL/6 mice, repressed in HEK293T cells following PTPRZ1 overexpression [54], and decreased in mice overexpressing PTPRZ1 [55]. PTPRZ1-dependent tyrosine phosphorylation of ErbB4 was also observed following PTN stimulation of mouse oligodendrocyte-lineage OL1 cells [50]. The functional PTPRZ1–ErbB4 interaction seems to be mediated by the scaffolding membrane-associated guanylate kinase, WW, and PDZ domain-containing (MAGI)-1 and MAGI-3 proteins that have been previously shown to bind to PTPRZ1 [56,57] through their PDZ4 domain [58]. ErbB4 seems to interact with the PDZ1 domain of the MAGI proteins [58]. PTPRZ1 has been shown to dephosphorylate tyrosine-phosphorylated MAGI-1 [53,57,59] and to decrease ErbB4-dependent tyrosine phosphorylation of MAGI-1 and MAGI-2 in H4 human neuroglioma cells [58].

G protein-coupled receptor kinase-interactor 1/Cool-associated tyrosine-phosphorylated 1 (GIT1/Cat-1) has been identified to bind to and be a PTPRZ1 substrate using the yeast substrate-trapping system. This interaction was verified in mammalian cells and the hippocampus and neocortex in the rat brain, and PTN increased tyrosine phosphorylation of GIT1/Cat-1 in B103 neuroblastoma [53,60] and mouse oligodendrocyte-lineage OL1 cells [50].

Paxillin has also been verified as a physiological PTPRZ1 substrate based on the typical substrate motif for the catalytic domain of PTPRZ1, which was deduced to be Glu/Asp-Glu/Asp-Glu/Asp-Xaa-Ile/Val-Tyr(P)-Xaa (Xaa is not an acidic residue). This substrate motif is present in the major phosphorylation site of paxillin at Tyr-118, and PTPRZ1 was found to efficiently dephosphorylate paxillin at this site in cells [53]. Tyrosine phosphorylation of paxillin was also increased following PTN treatment of mouse oligodendrocyte-lineage OL1 cells [50].

The neuronal tyrosine-phosphorylated phosphoinositide-3-kinase adaptor 2 (NYAP2) has also been suggested to be a PTPRZ1 substrate [53], and in support of this, PTN treatment of mouse oligodendrocyte-lineage OL1 cells increased the NYAP2 tyrosine phosphorylation [50]. However, it was not clarified whether the tyrosine phosphorylation of NYAP2 was a direct effect of PTPRZ1 TP activity or was mediated by other PTPRZ1-related TKs, such as Fyn [50,61]. Phosphorylated NYAPs interact with the phosphatidylinositol 3 kinase (PI3K) p85 subunit and activate PI3K, Akt, Rac1, and WAVE1 signaling [61], many of which have also been shown to be activated downstream of PTPRZ1, as discussed below.

Another adaptor protein involved in the activation of the PI3K-AKT pathway, actin filament-associated protein 1-like 2 (AFAP1L2/XB130), is also a PTPRZ1 substrate, as shown in cell-free assays as well as in HEK293T cells. In mouse oligodendrocyte-lineage OL1 cells, PTN induces AFAP1L2 tyrosine phosphorylation, and this is a prerequisite for the PTN-induced phosphorylation of Akt and mTOR and the oligodendrocyte progenitor cell differentiation [50].

Regulation of PI3K and/or Akt downstream of PTPRZ1 is one of the most extensively observed signaling pathways. In human embryonic stem cells, PTN activates Akt in a PTPRZ1-dependent manner, thus enhancing cell survival by inhibiting apoptosis [62]. In the same line, in GBM stem cells, PTN activates the PI3K/Akt pathway in a PTPRZ1- and Fyn-dependent manner [48]. PTN stimulates oligodendrocyte precursor cell differentiation partly through the PTPRZ1-dependent AFAP1L2 tyrosine phosphorylation, which then leads to the activation of the PI3K/AKT pathway [50]. In ovarian cancer cells, PTPRZ1 negatively regulates Akt kinase activity [63], in line with our data on mouse *Ptprz1*^−/−^ lung endothelial cells and tumors, that have increased levels of activated Akt compared to the corresponding wildtype [64]. In HUVEC, PTN and VEGFA_165_ activate PI3K downstream of PTPRZ1, and PI3K is required for both cell surface nucleolin localization [33,37] and cell migration [51].

In HUVEC, binding of PTN or VEGFA_165_ to PTPRZ1 leads to phosphorylation of β_3_ integrin on Tyr773 upstream of PI3K activation, cell surface nucleolin localization, and enhanced cell migration [30,33,37]. Although β_3_ integrin expression seems to be required for enhanced migration of endothelial cells by PTN [30], PTPRZ1- and PI3K-dependent, c-Src- and α_ν_β_3_-independent, and cyclin-dependent kinase 5 (CDK5) activation by PTN is also required for PTN-induced HUVEC migration [44]. Through the cell membrane functional complex of α_ν_β_3_ and PTPRZ1, PTN also activates xanthine oxidase to produce signaling levels of reactive oxygen species, required for PTN-induced endothelial and prostate cancer cell migration [65].

In triple-negative breast cancer cells, NFκΒ is activated downstream of PTPRZ1 following PTN binding, although the signaling that leads to NFκΒ activation is not clarified [15]. In HEK293 cells, transient transfection with either long or short PTPRZ1 isoforms has been shown to activate NFκΒ reporter gene transcription [66]. PTPRZ1 seems to negatively regulate the Rho-associated kinase (ROCK), and in *Ptprz1*-deficient mice, the aberrant activation of ROCK results in enhanced long-term potentiation and learning impairments in the animal [67]. There is also an observation that suggests that PTN targets N-cadherin for proteolysis through the ubiquitin-proteasome degradation pathway in a PTPRZ1-dependent manner [41], but this needs to be further and specifically studied. PTPRZ1 dephosphorylates voltage-gated sodium channels, and this modification positively shifts their voltage dependence, slows sodium channel inactivation, and increases the whole-cell sodium current [28].

PTPRZ1 has been shown to dephosphorylate anaplastic lymphoma kinase (ALK) autophosphorylation sites, an effect that is inhibited upon PTN binding to PTPRZ1 that leads to enhanced ALK tyrosine phosphorylation [68]. However, it has not been shown whether PTPRZ1 directly interacts with ALK, and we have been unable to detect such interaction in endothelial cells. Similarly, PTPRZ1 dephosphorylates the neurotrophins’ receptor TrkA, but not TrkB or TrkC, and attenuates TrkA activation induced by nerve growth factor in vivo. In the brain of *Ptprz1*^−/−^ mice, tyrosine phosphorylation of TrkA was significantly elevated [69].

**Figure 2:**
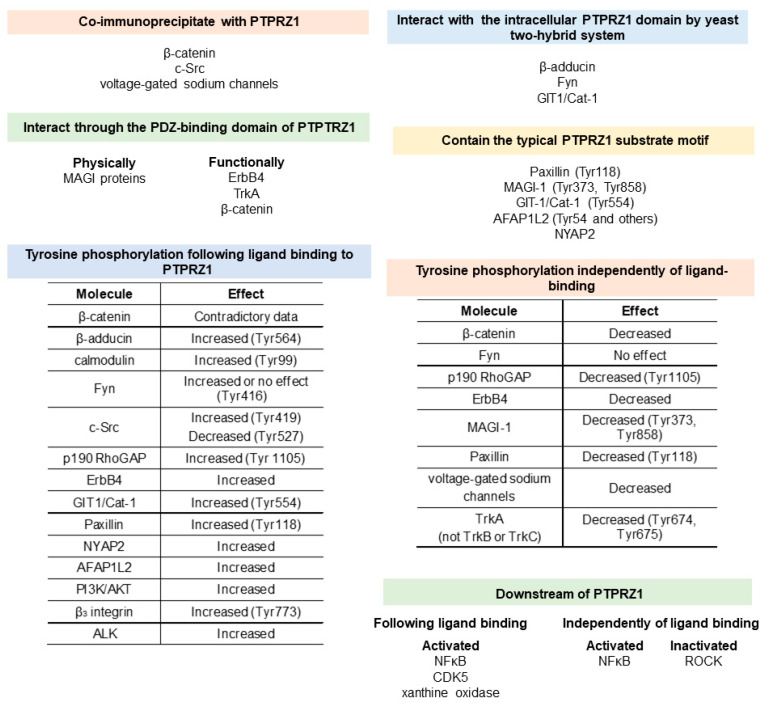
Signaling molecules that are regulated downstream of PTPRZ1. The experimental means of their identification are also illustrated. For more details, see the text.

Figure 2 illustrates the signaling molecules and how they have been identified downstream of PTPRZ1.

In most of the above-mentioned studies, PTPRZ1 is considered active in the absence of any of its soluble ligands, and it dephosphorylates its substrates. PTN binding to PTPRZ1 may cause PTPRZ1 dimerization, which inhibits the TP activity of the receptor, leading to enhanced tyrosine phosphorylation of its substrates, such as those mentioned above. This notion is based on early data that led to the ligand (PTN)-dependent receptor inactivation theory [40] and was supported by later data suggesting that in the absence of ligands, the negatively charged CS moieties prevent PTPRZ1 from clustering. The positively charged PTN neutralizes the electrostatic repulsion between CS chains, resulting in PTPRZ1 clustering [70]. However, this theory emphasizes the interaction of PTN with the CS moieties on PTPRZ1, although PTN may also interact with the protein core of the receptor, as discussed above. Moreover, PTN or other PTPRZ1 ligands may also act through the short PTPRZ1 transmembrane isoform that does not contain CS chains. Finally, not all PTPRZ1 ligands are positively charged, e.g., IL34. It has been shown that upon binding of PTN or VEGFA_165_ to PTPRZ1 in HUVEC, c-Src kinase is dephosphorylated in its carboxyterminal Tyr527, and this dephosphorylation is the first step for the activation of c-Src and its downstream signaling [30,37,51]. This observation suggests that PTN binding to PTPRZ1 may activate its TP activity, which through c-Src or other TKs leads to enhanced downstream tyrosine phosphorylation. This point needs further studies to be clarified, and depending on the PTPRZ1 isoform/glycosylation, it may be cell/tissue context-dependent.

## 6. PTPRZ1 in Cancer

The first study on the potential involvement of PTPRZ1 in tumor growth referred to the decreased expression of PTPRZ1 in lung adenocarcinomas compared to normal lung tissue, suggesting that PTPRZ1 may act as a tumor-suppressor [71]. In contrast, PTPRZ1 is highly expressed in small-cell lung carcinoma cells and human neuroendocrine tumor tissues, having an important oncogenic role in a mouse xenograft model of tumor progression [46]. Such a discrepancy has also been found in a more recent bioinformatic analysis, showing that PTPRZ1 expression is decreased in lung adenocarcinoma but increased in lung squamous cell carcinoma. In both cases, though, PTPRZ1 expression seems to be inversely correlated to either overall or disease-free survival [3].

Human gliomas are the most highly discussed tumors where PTPRZ1 is overexpressed and may regulate growth and invasion. An earlier study using mRNAs from 23 human glioma tissue samples showed that the transmembrane PTPRZ1 isoforms were expressed in all the lower-grade gliomas, but only in 45% of GBMs. Phosphacan was expressed in all grades [72]. However, in a subsequent study, PTPRZ1 protein was detected in the tumor cells, was elevated in astrocytic gliomas of different malignancy grades, and was associated with an increasing malignancy grade [73]. In a more recent study of the mRNA expression profiling of a series of clinical diffuse glioma samples of different grades, PTPRZ1 expression was found to be consistently upregulated in all glioma specimens, but it was significantly lower in GBM compared to lower-grade gliomas [74]. In GBM cells, PTPRZ1 mediates PTN-induced migration [74,75]. In the same line, PTPRZ1 mRNA was found enriched in GBM samples and positively regulated GBM cell migration [76]. Overexpression of either the long- or the short-transmembrane PTPRZ1 isoform in human U87MG GBM cells similarly enhanced cell migration and adhesion and activated NFκΒ-dependent signaling, suggesting that both isoforms may be valuable targets for GBM therapy [66]. Monoclonal antibodies that selectively bind to PTPRZ1 with low nanomolar affinities, coupled to the cytotoxin saporin, were shown to kill human U87MG GBM cells in vitro and delay the corresponding tumors’ growth in a mouse xenograft model in vivo [77]. Likewise, downregulation of PTPRZ1 expression by siRNA in human GBM U251MG cells injected subcutaneously into nude mice or in an orthotopic intracerebral model has resulted in significantly decreased tumor growth. In these cells, PTN-induced haptotaxis was decreased compared to cells expressing PTPRZ1 [78]. Using E98 GBM cells, knockdown of the PTPRZ-B isoform resulted in reduced migration and proliferation in vitro and inhibited orthotopic tumor growth in vivo [79].

In line with the high PTPRZ1 expression in embryonic stem cells, PTPRZ1 is preferentially expressed in GBM stem cells and mediates the stimulatory effects of PTN secreted by tumor-associated macrophages on GBM growth [48]. PTPRZ1 expression in GBM stem cells was shown to be upregulated by HOXA5 and contribute to cell survival and a worse GBM outcome [21]. Preferential PTPRZ1 expression in GBM stem cells correlates with that of the glycoprotein M6a (GPM6A) and targeting either of these molecules inhibited the invasive and sphere-forming ability of these cells and enhanced their sensitivity to radiation [80].

PTPRZ1 has also been included in a group of four genes comprising a gene signature that has been evaluated for the automation of the prediction of 35 brain tumors, distinguishing between GBM and meningioma cases [81]. This result requires further validation with a larger sample. Being considered a canonical cancer stem cell marker, PTPRZ1 has also been included in an integrative analysis of the heterogeneity present in GBM cancer stem cell populations by using a combination of flow cytometry and bulk and single-cell RNA-sequencing. A significant diversity of transcriptional profiles was observed between slow-cycling cells and cells expressing canonical cancer stem cell markers, with very little transcriptional overlapping [82].

An interesting observation is that the non-coding circular RNA for PTPRZ1 transcribed from the *Ptprz1* gene (hsa_circ_0133159) in a microRNA (MiR)-1261-dependent manner is highly expressed in gliomas and regulates the activation of P21-activated kinase 1 (PAK1), thus upregulating glioma cell proliferation and invasion [83]. A tumor atlas of primary GBMs created by a single-cell RNA-sequencing approach identified the outer radial glia-like cells, a cell-type population that undergoes a characteristic PTPRZ1-mediated mitotic translocation that promotes an invasive behavior [84]. The enhanced PTPRZ1 expression in GBM tissues is preserved in patient-derived tumorspheres compared to the normal human astrocytes, independently of the p53 mutation status [85].

Besides gliomas, PTPRZ1 is heterogeneously expressed in individual meningiomas and drives meningioma cell proliferation and tumorigenesis [86]. It is also the fifth most frequently occurring gene in head and neck cancer, as suggested by analyzing a large cohort of single-cell transcriptomics data [87], although the functional significance of this observation remains to be studied. In oral cavity squamous cell carcinoma, PTPRZ1 was found by immunohistochemistry to be expressed more frequently in lower-grade tumors and was associated with improved patient survival [88]. On the other hand, PTPRZ1 overexpression promotes oral submucous fibrosis, a potentially malignant disease of the oral cavity, through the nuclear translocation of β-catenin [43].

In stomach adenocarcinoma, positive PTPRZ1 immunohistochemical reactivity has been observed and has been associated with gastric cancer progression [89]. However, PTPRZ1 mRNA levels are significantly lower in gastric adenocarcinoma compared to the corresponding normal tissue [3].

PTPRZ1 mRNA levels are decreased in colorectal cancers compared to those in adjacent normal mucosae [90]. In a promoter methylation analysis of 131 surgical specimens obtained from patients with sporadic colorectal cancers, the *Ptprz1* promoter was found hypermethylated in tumor cells compared to the corresponding adjacent normal tissue [91], supporting a decreased PTPRZ1 expression in colorectal cancer. In a following study that involved 102 colorectal cancer tissues, amplification in the region containing the *Ptprz1* gene was observed in 20% of cases [92]. By analyzing the mRNA levels of PTPRZ1 in 16 tissues obtained from patients with colorectal carcinoma, no significant difference in the *Ptprz1* gene expression was found compared to the corresponding normal tissues [93]; similarly, the protein PTPRZ1 levels were not found to be different between 25 colorectal carcinoma and 5 normal tissues, studied by immunohistochemistry and Western blot analysis [94]. In a recent bioinformatic analysis, PTPRZ1 mRNA levels were significantly decreased in both colon and rectum adenocarcinomas, and this decrease may be associated with overall and/or disease-free survival [3].

PTPRZ1 was found by immunohistochemistry to be overexpressed in human primary and metastatic melanomas, but not in the melanocytes of healthy skin [95]. However, mRNA levels of PTPRZ1 were not found significantly different between normal and tumor tissues in skin cutaneous melanoma [3]. In uveal melanoma cells, PTPRZ1 is overexpressed and positively affects proliferation and invasion in vitro [39].

PTPRZ1 is expressed in different types of human breast cancers, both in the breast cancer cells themselves and in carcinoma-associated fibroblasts. PTPRZ1 protein has been detected by immunohistochemistry in the cell membrane, the cytoplasm, and the nucleus in different breast cancer cells [96], in line with another study showing cytoplasmic and nuclear PTPRZ1 localization in endothelial cells [33]. In triple-negative breast cancer, PTPRZ1 is overexpressed, as assessed by immunohistochemistry in 325 cases of breast cancer, and it may be an independent risk indicator for recurrence and metastasis [97]. Using microarray data from the GEPIA database, PTPRZ1 was found significantly downregulated in breast cancer samples derived from patients that received no chemotherapy compared to samples from the normal group. PTPRZ1 expression was found to be significantly upregulated following chemotherapy, based on data from the GEO database. Doxorubicin enhanced both PTPRZ1 and PTN expression in the triple-negative breast cancer MDA-MB-231 and MDA-MB-453 cells, promoting cell proliferation and inhibiting apoptosis through PTPRZ1-dependent activation of NFκΒ [15]. High PTPRZ1 expression seems to correlate with a negative effect on overall patient survival [3]. In a study that analyzed the differential RNA expression patterns between breast cancer with and without bone metastasis, using 1091 primary breast cancer samples included in The Cancer Genome Atlas database, a significant correlation was found between PTPRZ1 expression and the survival rate in breast cancer patients with bone metastasis [98].

In cervical carcinoma, PTPRZ1 expression was found significantly higher compared to the normal cervical epithelium, significantly higher in patients with smaller (≤2 cm) compared to those with larger (>2 cm) tumor sizes, and higher in squamous cell carcinoma than in adenocarcinoma [99]. In a bioinformatic analysis, PTPRZ1 was also found to be upregulated in cervical squamous cell carcinoma and endocervical adenocarcinoma and might be associated with better overall and disease-free survival [3].

PTPRZ1 is expressed in epithelial ovarian cancer cells and enhances cell viability through the inhibition of apoptosis. It was also found upregulated in serous ovarian tumor tissue relative to normal ovarian surface epithelial tissue [100]. However, PTPRZ1 mRNA levels were found to be significantly downregulated in ovarian serous cystadenocarcinoma [3], in line with a recent study using transcriptomic data, showing an abnormally low PTPRZ1 expression in ovarian cancer tissues and in cisplatin-resistant ovarian cancer cells. Overexpression of PTPRZ1 enhanced the sensitivity of ovarian cancer cells to cisplatin and enhanced cell apoptosis in vitro, and inhibited tumor growth and resistance to cisplatin in vivo [63].

In prostate cancer, PTPRZ1 mRNA levels are significantly decreased compared to normal prostate tissue [3]. This is in line with data showing that downregulation of PTPRZ1 expression in human prostate DU145 and PC3 cells initiated EMT and enhanced prostate cancer cell migration and invasion in vitro, and metastasis in vivo [101].

In renal cell carcinoma cell lines, downregulation of PTPRZ1 expression by siRNA has been shown to decrease the amounts of nuclear β-catenin, decrease the expression of target genes, and suppress cell proliferation [16]. In contrast, PTPRZ1 is overexpressed in renal cell carcinomas following the loss of VHL activation, activates β-catenin, and enhances cell proliferation [19]. However, PTPRZ1 mRNA levels are decreased in renal cell carcinomas [3].

In a sample of 30 osteosarcoma patients, the *Ptprz1* gene was overexpressed in 73% and under-expressed in 27% of cases. There was no correlation between the *Ptprz1* gene expression profile, clinicopathological parameters, and survival [102]. However, in a later study using *Ptprz1^−/−^* 129SV/Ev Trp53-heterozygous mice, it was found that *Ptprz1* gene deletion enhanced osteosarcoma development, characterized by enhanced tyrosine phosphorylation and cell proliferation, suggesting that PTPRZ1 acts as a tumor-suppressor for osteosarcoma [103].

PTPRZ1 expression is also increased in lymphoma tissues from patients with diffuse large B lymphoma, especially of high risk, correlates with the proportion of tumor-associated macrophages that promote lymphoma growth, and coincides with an increased proportion of cancer stem cells [104].

A summary of the data related to PTPRZ1 expression and its role in different cancer types is presented in Table 1.

## 7. PTPRZ1 Fusion Proteins in Cancer

To identify driver fusion proteins in GBM, RNA-sequencing of 272 gliomas identified PTPRZ1-MET (ZM) fusion transcripts only in grade III astrocytomas or secondary GBMs. The fusion transcripts result in a protein that has a molecular mass of 145 kDa, which is difficult to distinguish from wildtype MET [105,106,107]. The ZM fusion protein forms homodimers or heterodimers with wildtype MET, leading to enhanced MET tyrosine phosphorylation levels in the absence of its ligand, hepatocyte growth factor, although the latter can further activate ZM autophosphorylation [108]. The ZM fusion protein has been described to contain sequences encoding the carbonic anhydrase and the fibronectin type III domain of PTPRZ1 fused to the dimerization domain, immunoglobulin-like domains, transmembrane domain, and the tyrosine kinase domain of MET [108], or to result from the highly active promoter of the *Ptprz1* gene fused to exons 2–21 of *Met*, leading to overexpressed MET and activated downstream signaling [109,110]. In all cases, GBMs harboring a ZM fusion exhibit a more aggressive phenotype and are associated with a poor patient prognosis [108]. PTPRZ1-MET fusion proteins are also found in pediatric GBMs with enhanced MET expression and activity, sensitive to MET inhibitors [109,110]. Exosomes from GBM cells harboring the ZM fusion have higher MET expression and activity compared to those from non-ZM fusion GBM cells, and they induce EMT when they are transferred to non-ZM fusion GBM cells or normal human astrocytes [111]. ZM fusion has also been detected in a small number of brain metastases of lung cancer [112].

In spitzoid neoplasms, the PTPRZ1-NFAM1 fusion gene has been identified in two patients, associated with a copy gain in the kinase fusion gene. NFAT-activated protein 1, which is the protein encoded by *Nfam1*, is a transmembrane receptor that regulates cytokine production and is mostly expressed in immune cells [113].

Another fusion transcript, PTPRZ1-ETV1, has been more recently identified in 6% of the tested gliomas, including GBMs, one anaplastic oligodendroglioma, and one pilocytic astrocytoma. This fusion consists of the *Ptprz1* promoter in frame with the highly conserved DNA-binding domain of the ETV1 transcription factor. The latter is a member of the ETS family of transcription factors, known as oncogenic drivers in different types of tumors [114]. To date, this fusion protein’s prognostic or therapeutic value is unknown.

More recently, PTPRZ1 has been identified as a BRAF fusion partner in juvenile pilocytic astrocytomas, but information on the functional significance of this fusion protein is missing [115].

## 8. PTPRZ1 in Angiogenesis

Single-cell RNA-sequencing data from different organs in the Atlas Cancer database show that PTPRZ1 is not significantly expressed in endothelial cells in any organ. PTPRZ1 has not been detected either in cerebral microvascular endothelial cells isolated from small, freshly obtained specimens of adult normal brain adherent to human arteriovenous malformations, and the stimulatory effects of PTN in these cells have been attributed to a currently unknown receptor [73]. However, it is expressed in HUVEC [33,37,51,116] and microvascular endothelial cells isolated from mouse lungs [64] and is required for both PTN- [51,116] and VEGFA_165_-induced [37] endothelial cell migration and tube formation in vitro. Expression of α_ν_β_3_ integrin seems to be a prerequisite for the stimulatory effects of PTN and VEGFA through PTPRZ1 on endothelial cell migration [30,37], and the mechanisms involved are being investigated.

Data on the role of PTPRZ1 in angiogenesis in vivo are limited. SV129/B6 mice bearing a constitutive deletion of *Ptprz1* display significantly more bone marrow hematopoietic stem cells compared to the corresponding wildtype mice [117] and increased angiogenesis in the heart [118] and lungs [64]. Further studies are in progress.

## 9. Pharmacological Targeting of PTPRZ1 in Cancer

TP inhibitors are not extensively exploited, and no such drugs exist in the clinic to date. The first small molecule that selectively and potently inhibits the catalytic activity of PTPRZ1 was found by in vitro screening of a chemical library and was named SCB4380. This molecule inhibited rat C6 glioma cell proliferation and migration in vitro and inhibited tumor growth in vivo, favoring the notion that selective inhibition of PTPRZ1 may be a promising therapeutic approach in GBM [119]. In the same line, another cell-permeable small-molecule inhibitor of PTPRZ1, NAZ2329, was found to decrease the expression of the stem cell transcription factor SOX2 in rat C6 and human U251 GBM cells, thus inhibiting their growth in vitro and in vivo. Even more, NAZ2329 and the alkylating agent temozolomide have a synergistic effect in decreasing GBM cell growth [120]. A few more blood–brain barrier-permeable small molecules that selectively interact with the intracellular D1 domain of PTPRZ1 and inhibit its TP activity were designed and found to increase the tyrosine phosphorylation of known PTPRZ1 substrates [121]. The effect of these latter compounds on angiogenesis and cancer growth is being tested.

Besides small molecules that target the TP activity of PTPRZ1, another approach is to develop molecules that would inhibit ligand binding to the extracellular PRPRZ1 domains. Early studies in neurons have shown that polyclonal antibodies against the extracellular domain of PTPRZ1 suppress PTN-induced neuronal migration [122]. Antibodies targeting the extracellular domain of the short PTPRZ1 isoform have also been shown to modestly delay GBM growth in mice in vivo. When such antibodies were coupled to a cytotoxin, they killed human U87MG GBM cells in vitro and significantly delayed GBM growth in a mouse xenograft model [77]. More recently, it was shown that an antibody against the extracellular PTPRZ1 domain in GBM stem cells inhibits PTN binding and thus suppresses GBM growth in mice, leading to prolonged survival [48].

PTPRZ1 has also been identified as one of the GBM-associated antigens that could be considered a target for immunotherapy in both HLA-A 02-positive [123] and negative [124] GBM. The PTPRZ1 peptides, together with the rest of the HLA-A2-restricted tumor-associated antigens identified by peptidomics, have been used to formulate a multi-peptide vaccine (IMA950) that has entered phase I/II clinical trials for gliomas and showed spontaneous antigen-specific T-cell responses that were better in grade II and III compared to GBM patients [125]. Using an in-silico approach, another PTPRZ1 domain was found to induce the host’s B- and T-cell immune response against GBM and was fused with domains from other proteins to construct and characterize a multi-domain recombinant vaccine that will be validated by further in vitro and in vivo experimental studies [126].

A more recent approach developed for the treatment of highly aggressive uveal melanoma was the construction of indocyanine green-labeled manganese metal-organic framework nanoparticles that carry siRNA for the lncRNA OUM1 and PTPRZ1, together with cisplatin. Nanoparticles were linked with the RGD peptide for targeting and were shown to kill uveal melanoma in vitro and delay tumor growth in vivo through selective siRNA knockdown and enhanced cisplatin cytotoxicity [39].

Besides developing drugs that target PTPRZ1 to inhibit its signaling, PTPRZ1 has also been considered as a cell membrane molecule exploited to target GBM stem cells with cytotoxic chemotherapeutics. In a study using patient-derived GBM tissues cultured in a microchannel network chip, resistance to temozolomide and radiation is observed, similar to what is observed in GBM patients, and can be overcome by nanovesicles displaying an anti-PTPRZ1 peptide and loaded with temozolomide [127]. In a similar approach, a self-assembled hybrid micelle that can cross the blood–brain barrier, and simultaneously target M2-like tumor-associated macrophages by a specific peptide and GBM stem cells by an anti-PTPRZ1 antibody, was developed as a nanocarrier to deliver the chemotherapeutic agent doxorubicin to the GBM tissue. This nanocarrier was shown to be effective in reshaping the immune microenvironment and decreasing the growth and the invasive potential of GBM stem cells [128]. One drawback of this latter study is that the anti-PTPRZ1 antibody used targets the intracellular TP domain of PTPRZ1, and it is not clear or shown how this antibody targets GBM stem cells.

Finally, a recent study that used single-cell RNA-sequencing datasets from 37 GBM patients to look for GBM stem-like marker candidates identified PTPRZ1 as one of the most highly expressed surface markers to be used for GBM stem cell isolation and identification [129].

## 10. Concerns and Future Directions

During the last few years, there has been an increasing interest in the possible implication of PTPRZ1 in several malignancies, with most of the studies focusing on gliomas, in line with PTPRZ1 expression predominantly in the adult brain. However, some points need to be further clarified to guide proper PTPRZ1 exploitation for the development of therapeutics and/or as a biomarker. These points can be summarized as follows:

(a) Expression of different PTPRZ1 isoforms and the functional significance in each case have not been extensively elucidated. Taking into account that glycosylation of PTPRZ1 can be different in different organs or change during development or pathologies, even in the same organ, it becomes even more complicated to precisely identify the exact PTPRZ1 structure involved in any given function/pathology.
(b) PTPRZ1 can be cleaved by proteolytic enzymes, such as plasmin [130] or metalloproteinases and secretases [131]. Plasmin cleaves PTPRZ1 at multiple sites of the extracellular domain, and the proteolytic fragments are present in the normal brain, suggesting that PTPRZ1 processing occurs in vivo. Similarly, cleavage of the extracellular juxtamembrane region of PTPRZ1 by TNFα-converting enzyme and matrix metalloproteinase 9 physiologically occurs in the brain, and the remaining membrane-tethered fragment is cleaved by presenilin/γ-secretase, releasing its intracellular region into the cytoplasm. Whether and how this processing affects the implication of PTPRZ1 in cancer growth and metastasis and/or angiogenesis has never been addressed.
(c) Very few pharmacological tools have been developed to inhibit PTPRZ1 downstream signaling. A small number of selective PTPRZ1 TP inhibitors have been developed, as discussed above, and have been shown to inhibit GBM cell growth. However, these studies are limited and have employed only one or two GBM cell lines. Considering the great heterogeneity of GBM cells, it is not safe to conclude from these few studies. This hesitation is further supported by the prevailing notion that PTN binds to PTPRZ1 and inhibits its TP activity, while at the same time, it has a stimulatory effect on GBM growth and angiogenesis, which contradicts the efficacy of the TP inhibitors. However, there are also studies showing a negative effect of PTN in GBM cell growth and migration [8,132], and we have shown that the stimulatory or inhibitory effect in GBM cell migration depends on the expression of α_ν_β_3_ integrin [30], suggesting that the tumor microenvironment should be considered in each case, and identification of proper biomarkers should precede the use of PTPRZ1 TP inhibitors.
(d) It has not become clear whether the best targeting strategy is to inhibit PTPRZ1 TP activity or to inhibit ligand binding to its extracellular domain. Data show that inhibition of PTN binding to PTPRZ1 by an antibody that targets the extracellular PTPRZ1 domain inhibits GBM growth [48]. This approach is also supported by an older study showing that in glioma cells that were made to not express PTPRZ1, expression of only the PTPRZ-B extracellular domain is sufficient for cell migration, while the C-terminal PDZ-binding domain is required for proliferation [79].
(e) PTPRZ1 is upregulated in some cancer types and downregulated in others, while these changes do not coincide with overall or disease-free survival. For example, PTPRZ1 is downregulated in lung adenocarcinoma but upregulated in lung squamous cell carcinoma. In both types of lung cancer, PTPRZ1 expression inversely correlates with overall or disease-free survival. In other types of cancer, such as GBM or uveal melanoma, PTPRZ1 expression positively correlates with overall or disease-free survival, while in others there is no association, despite the altered expression [3]. Identification of the PTPRZ1 downstream signaling pathways in the presence or absence of its ligands should help explain these opposing effects, that could be due to the diverse tumor microenvironments.
(f) An interesting loop that needs to be further validated comes from the data showing that NFκΒ binds to and activates the PTPRZ1 promoter [20], while it is also activated downstream of PTPRZ1 [15,65]. NFκΒ has a major role in the regulation of inflammation and cancer progression [133], and the validity of PTPRZ1 as a target to inhibit NFκΒ signaling in cancer deserves further investigation.
(g) PTPRZ1 has multiple soluble ligands that affect cancer angiogenesis, such as PTN, FGF2, and VEGFA, suggesting that it may serve as an important homeostatic pathway to help prevent excess angiogenesis. This is supported by the observation that in PTPRZ1 knockout mice, heart angiogenesis is significantly enhanced [118]. The observation that PTPRZ1 is required for significant VEGFA-induced angiogenic endothelial cell functions [37,38] needs to be further exploited to identify whether and how the signaling pathways downstream of VEGF receptors and PTPRZ1 crosstalk and regulate angiogenesis. It also leads to the hypothesis that PTPRZ1 may mediate the effects of FGF2 or VEGFA on cancer cells, even in the absence of their specific receptors.
(h) Although PTPRZ1 is expressed by endothelial cells in vitro, to date, it has not been detected in mature blood vessels in vivo. This may be due to the quiescent state of endothelial cells in the already formed blood vessels in the tissues, while endothelial cells in culture mimic the state of the endothelial cells during active angiogenesis when they proliferate and migrate to form the new vessels. This point needs to be further addressed to validate PTPRZ1 as a target for anti-angiogenic approaches.
(i) Expression of PTPRZ1 is directly regulated by HIF2, as discussed above. HIF2 expression is tissue-specific, primarily observed in endothelial cells, but also in adipocytes, smooth-muscle cells, trophoblasts, and lung epithelial cells. HIF2 in endothelial cells controls the transcription of numerous genes involved in the regulation of angiogenesis [134], further supporting the notion that PTPRZ1 may be an angiogenesis regulator.
(j) An interesting hypothesis comes from recent data showing that anti-tumor B-cell responses may have a significant role in opposing the development of lung adenocarcinoma and breast cancer and enhancing their response to immune checkpoint blockade [135,136]. In *Ptprz1*^−/−^ mice, the number of mature B-cells is decreased [137], raising the hypothesis that this might be a mechanism through which PTPRZ1 affects cancer development depending on the inherent immunogenicity of each tumor. It is also of interest to note that MK enhances B-cells’ survival through PTPRZ1 [137], an observation that, in combination with the decreased number of mature B-cells in *Ptprz1*^−/−^ mice, opposes the model that supports dimerization-induced PTPRZ1 TP inactivation following ligand binding.

In conclusion, there is an increasing interest in PTPRZ1′s involvement in angiogenesis and cancer and its potential importance in the regulation of processes that drive carcinogenesis and/or cancer growth and invasion. The potential therapeutic benefit of targeting PTPRZ1 will most likely be context-dependent, and future efforts should be focused on the elucidation of the interactions of PTPRZ1 with other cell surface receptors and the signaling pathways activated downstream of PTPRZ1 in the presence or absence of its ligands, elucidating the involvement of the different PTPRZ1 domains. PTPRZ1-based therapeutics could help overcome resistance to existing therapies and could be used in combination with chemotherapy or other targeted therapies, depending on the tumor type. PTPRZ1 may also prove to be a valuable biomarker for the choice of the best therapeutic protocol or to be used for selective targeting of cancer cells that overexpress it.

## Figures and Tables

**Table 1:** PTPRZ1 expression and suggested role(s) in various cancers.

Cancer Type	PTPRZ1 Expression	Role	References
Lung adenocarcinoma	Decreased	Not studied	[3,71]
Small-cell lung carcinoma	Increased	Enhances tumor progression	[46]
Lung squamous cell carcinoma	Increased	Not studied	[3]
Gliomas/GBM	Increased, especially in GBM stem cells	Enhances growth, invasion, stem cell survival, and migration	[21,48,66,72,73,74,75,76,77,78,79,80]
Meningioma	Increased	Drives cell proliferation and tumorigenesis	[86]
Oral cavity squamous cell carcinoma	Expressed in lower-grade tumors	Improves patient survival	[88]
Stomach adenocarcinoma	Decreased	Relates to cancer progression	[3,89]
Colorectal cancer	Decreased	Not studied	[3,90,91,92,93,94]
Melanoma	Increased	Positively regulates proliferation and invasion	[39]
Breast cancer	Increased in triple-negativeDecreased in invasive cancer	Not studied	[97]
Cervical carcinoma	Increased	Not studied	[99]
Epithelial ovarian cancer	Decreased	Inversely correlates with resistance to chemotherapy and tumor growth	[63,100]
Prostate cancer	Decreased	Negatively regulates cell migration, invasion, and metastasis	[3,101]
Renal cell carcinoma	Contradictory data	Activates β-catenin and enhances cell proliferation	[3,16,19]
Osteosarcoma	Overexpressed in 73% and under-expressed in 27% of cases	*Ptprz1* gene deletion enhances cell proliferation	[102,103]
Diffuse large B-cell lymphoma	Increased	Enhances lymphoma growth	[104]
